# Specialized Smartphone Intervention Apps: Review of 2014 to 2018 NIH Funded Grants

**DOI:** 10.2196/14655

**Published:** 2019-07-29

**Authors:** William B Hansen, Lawrence M Scheier

**Affiliations:** 1 Prevention Strategies, LLC Brown Summit, NC United States; 2 LARS Research Institute Scottsdale, AZ United States

**Keywords:** smartphone, intervention, funded grants, mobile phone

## Abstract

**Background:**

The widespread adoption of smartphones provides researchers with expanded opportunities for developing, testing and implementing interventions. National Institutes of Health (NIH) funds competitive, investigator-initiated grant applications. Funded grants represent the state of the science and therefore are expected to anticipate the progression of research in the near future.

**Objective:**

The objective of this paper is to provide an analysis of the kinds of smartphone-based intervention apps funded in NIH research grants during the five-year period between 2014 and 2018.

**Methods:**

We queried NIH Reporter to identify candidate funded grants that addressed mHealth and the use of smartphones. From 1524 potential grants, we identified 397 that met the requisites of including an intervention app. Each grant’s abstract was analyzed to understand the focus of intervention. The year of funding, type of activity (eg, R01, R34, and so on) and funding were noted.

**Results:**

We identified 13 categories of strategies employed in funded smartphone intervention apps. Most grants included either one (35.0%) or two (39.0%) intervention approaches. These included artificial intelligence (57 apps), bionic adaptation (33 apps), cognitive and behavioral therapies (68 apps), contingency management (24 apps), education and information (85 apps), enhanced motivation (50 apps), facilitating, reminding and referring (60 apps), gaming and gamification (52 apps), mindfulness training (18 apps), monitoring and feedback (192 apps), norm setting (7 apps), skills training (85 apps) and social support and social networking (59 apps). The most frequently observed grant types included Small Business Innovation Research (SBIR) and Small Business Technology Transfer (STTR) grants (40.8%) and Research Project Grants (R01s) (26.2%). The number of grants funded increased through the five-year period from 60 in 2014 to 112 in 2018.

**Conclusions:**

Smartphone intervention apps are increasingly competitive for NIH funding. They reflect a wide diversity of approaches that have significant potential for use in applied settings.

## Introduction

### Public Health Interventions

Through its various federal agencies, the US government has evolved a broad public health mandate to prevent disease and improve health. Part and parcel of this mandate is to find creative and innovative ways to enhance the public’s health and reduce the burdens of disease and disability. In recent years, the National Institutes of Health (NIH) and its 27 Institutes and Centers have searched for promising ways to capitalize on the public’s use of mobile phone technology to service its mission. Otherwise known as mobile health (mHealth), the use of cellular and smartphone technology has provided a platform to extend the reach of health promotion and disease prevention activities. We differentiate mHealth technology, which is defined as “mobile communications for health information and services” from electronic health (eHealth), the latter which primarily involves the use of the Internet for delivery of behavioral and health-related interventions [1-4]. Mobile communication can include, but not be limited to, smartphones, tablets, patient monitoring devices, personal digital assistants, and bodily or household sensors that rely on wireless communication. Given the widespread presence of mobile technology, potential users are no longer bound by periodically showing up for clinic visits and the inconvenience of waiting rooms and stalled scheduling. The monitoring and reporting of behavior, tracking events that precipitate disease conditions, or monitoring adherence can all be conducted in real-time, and a wide assortment of pertinent information can be transported electronically to medical records or even stored in comprehensive research databases.

Several systematic reviews and meta-analyses have reinforced the utility of mobile phones and smartphones (including wireless hand-held tablet devices and cellular phones) in a variety of health domains. These include application of mHealth technology in care delivery and chronic disease management [5,6] with specific applications monitoring hypertension [7,8], medication adherence support [9], obesity and weight loss [10], diabetes mellitus [11], substance use prevention [12,13], mental health [14-16] and physical activity [17,18], to name a few. Apps have also been developed for asthma self-care management [19], smoking cessation [20], maternal and neonatal care [21], and stress reduction using mindfulness strategies [22], all of which include provision of biofeedback [23]. Additional reviews have focused more exclusively on mobile apps targeting diseases identified by the WHO as major global health priorities [24]. For the most part, these reviews support promising results with potential healthcare benefits for a wide selection of evidence-based mobile and smartphone app interventions.

There is a considerable upside to the plethora of mobile phone apps now available. According to the trade organization GSMA Intelligence, which tracks trends in mobile phone usage worldwide, cell phone use worldwide has topped five billion, with roughly 67% market penetration and 3.2% annual projected growth [25]. In the United States alone, there are over 262 million mobile phone subscribers [25]. Increases in mobile phone usage are slated for the traditionally developing or low-income countries where expansion of mobile phone infrastructure (ie, 4G networks, cell transmission towers, and LTE broadband transmission) is taking place [26]. Using digital distribution platforms like Apple’s AppStore for iOS products or Google’s Google Play for Android products, approximately 1500 apps are downloaded daily with 178 billion downloaded in 2017 and 205 billion in 2018 [27]. A mobile app industry tracking company reports there are 318,000 health-related mobile phone apps with approximately 200 new ones added daily [28]. Based on Apple Store statistics, health and fitness apps represented 3.0% of the total downloads, lifestyle represented 8.3% of the downloaded apps, medical was 1.8% and the leading app download category was games at 24.9%.

More importantly, availability of free and low-cost health-related applications has dramatically increased as well. FitBit, a digital healthcare company, is the leading fitness tracking app among those labeled as wireless-enabled wearable sensor technology and that are used specifically for monitoring health and vitals. The free, downloadable app monitors health and vitals like heart rate, pulse, sleep quality, and calories burned, and can both accurately measure steps using a pedometer, as well as monitor activity through an accelerometer-based motion sensor. A new sector is growing to handle digital biomarkers that can instantly regulate a patient’s insulin intake or monitor their breathing capacity (ie, smart inhalers) and report this to clinical providers. The availability of apps in general as well as the increased market for health-related apps all speaks volumes regarding demand. One barometer of this demand is revenue from global mobile app sales. In 2014, revenues from mobile app sales hovered around 35 billion US dollars. In 2018, these sales skyrocketed to 92 billion US dollars [29] with the duo of Samsung and Apple sharing the lion’s share of revenues at 50%. This number is projected to reach 188.9 billion US dollars in 2020.

The use of mobile phone apps for healthcare piggybacks onto several emerging trends. Smartphone sales have skyrocketed in the past decade, and with their advanced features they now practically function like a handheld computer. This is due to improved technological capabilities which include wireless connectivity allowing Internet access, faster chip processors and download speeds, longer battery life, finer digital pixilation, and multimedia graphic imaging capability, all at a lower cost to subscribers. Freeware apps like Skype, Fring, and iCall utilize Voice of Internet Protocol (VoIP) to achieve real-time synchronous remote teleconferencing (ie, video chat). The global positioning system (GPS) capability enables remote location monitoring, making it possible to monitor disease vectors as part of public health surveillance [30,31], to track physical activity in cardiac rehabilitation [32] and to find dementia patients who wander outside of care facilities [33].

Smartphone camera technology has improved dramatically, and with higher resolution digital imaging it can now enable bio-optical sensing and real-time synchronous sharing of pictures of patients’ skin with their dermatologist or sharing of medical conditions with healthcare staff for diagnosis, treatment and remediation. Connectivity to cloud computing allows physicians, for example, to exchange laboratory or radiographic images for medical consultations using servers that are compliant with data privacy regulations [15]. More and more, even in resource limited countries, mobile technology provides a bridge to healthcare connectivity, making it possible for patients (or their families) to receive educational materials, monitor their health, track symptoms or conduct self-assessments [34].

Smartphone apps allow users to connect with their healthcare provider and share patient experiences as well as receive individualized instruction by transmitting data in real-time. This may help individuals overcome resource limitations that may arise from the large distances between their residence and their provider (access to care) and structural barriers that arise from cultural or behavioral factors that limit care provision. There are now numerous projects underway supporting public health surveillance in Sub-Saharan Africa that are leveraging mobile health-related apps [35,36] to increase treatment adherence, improve delivery of care [30], and monitor communicable diseases (ie, malaria) and other health problems (eg, child malnutrition). Even physician providers themselves are using mobile apps to learn about care delivery in remote areas of Africa [37], and this is part of an ongoing, worldwide trend for health care professionals who require greater access to point of care tools for decision making [38]. The burgeoning use of mobile phones for provision of healthcare is coupled with what is termed lab-on-a-chip technology that allows biochemical and diagnostic assays (eg, blood glucose monitoring or cholesterol testing) to be performed with sweat, saliva, urine or blood using the smartphone in vivo [39]. In all these examples, the portability, ease of use, flexibility, customization, convenience, and privacy of information was highly attractive to users.

### Missing Pieces of the Mobile Phone Healthcare Puzzle

What is missing from this picture, however, is a deeper understanding of the organization of mobile apps, in particular whether there are common themes underlying mobile app intervention strategies (ie, treatment adherence, behavior modification, appointment reminders, and data collection using self-reporting or remote bio-sensors for symptom tracking), service delivery strategies (ie, platforms used to deliver interventions), the medical and behavioral conditions that apps emphasize, their intervention goals, integration with other service platforms (ie, web or in person), and characterization of the target populations, all of which can help clarify the event horizon. To our knowledge, this type of summarization has not been previously conducted, with the few research-based systematic reviews of mHealth applications highly topic dependent (ie, focusing on at most one disease or health focus with a specific target population). It is particularly relevant to understand the US government’s scientific research investment portfolio, which through various grant mechanisms supports app development that specifically addresses the nation’s stated public health agenda [40]. Therefore, in the current study, we undertake the synthesizing of NIH-funded mobile phone apps between 2014 and 2018 based on a set of organized criteria that will inform the future of public health initiatives using mHealth strategies.

## Methods

### Sample

We searched NIH Reporter [41] using the following key terms in the text search field: (1) *mHealth* or *smartphone* or *mobile* or *phone* or *Android* or *iOS* or *game* or *gaming* or *gamification* and (2) *intervention* or *treatment* or *randomized* or *RCT* or *program*. We limited the search period to the five fiscal years of 2014 through 2018. At times, insufficient information was available on the NIH Reporter that made it hard to detect whether an app was being used in the research. This prompted us to widen the search engines (Google) and to include Grantome.com (an independent resource for searching for grant awards) as a means of locating additional information. In some cases, we used Google Scholar to track down publications, when available, that could clarify the research focus and utilization of a true app.

We completed this search for each of the following agencies:

1. National Institute on Alcohol Abuse & Alcoholism (NIAAA)
2. National Institute on Aging (NIA)
3. National Institute of Allergy and Infectious Diseases (NIAID)
4. National Institute of Arthritis and Musculoskeletal and Skin Diseases (NIAMS)
5. National Institute on Drug Abuse (NIDA)
6. National Institute on Deafness and other Communication Disorders (NIDCD)
7. National Institute of Dental and Craniofacial Research (NIDCR)
8. National Institute of Diabetes and Digestive and Kidney Diseases (NIDDK)
9. National Institute of Biomedical Imaging and Bioengineering (NIBIB)
10. National Institute of Environmental and Health Sciences (NIEHS)
11. National Eye Institute (NEI)
12. National Institute of General Medicine Sciences (NIGMS)
13. National Institute of Child Health and Human Development (NICHD)
14. National Human Genome Research Institute (NHGRI)
15. National Heart, Lung, and Blood Institute (NHLBI)
16. National Library of Medicine (NLM)
17. National Institute on Minority Health and Health Disparities (NIMHD)
18. National Institute of Mental Health (NIMH)
19. National Institute of Nursing Research (NINR)
20. National Institute of Neurological Disorders and Stroke (NINDS)

For each funded grant, we retrieved the abstract, title, start date, activity code, and first year funding amount. We subsequently retained only the following grant activity codes: R01, R03, R15, R21, R34, R35, R41, R42, R43, R44, R56 and R61. We excluded all D, F, K, O, P, S, U and Z awards as well as R13, R25, R36, R37 and R90 awards. This left us with the bulk of peer reviewed research grant mechanisms that included technology-related research [42].

### Initial Documentation

For each grant we reviewed the abstract, the only publicly available description of the project that might elucidate the content and methods. We first eliminated grants that did not have a planned intervention. We also eliminated grants that only used standard features of smartphones, such as SMS or text messaging, although if there were added features that were novel in any way those grants were retained.

We read through each abstract and provided brief summaries about: (1) the topic of the app (eg, the specific disease or behavior addressed); (2) the specific strategy employed by the app (ie, how the app was to be used for intervention); (3) the intended outcome (ie, goals of the intervention); (4) whether the app was applied to a standalone intervention or used in an ancillary way in conjunction with other forms of care; (5) the age group targeted by the app; (6) the population on which it would be tested or used; and (7) the name of the app.

### Categorization Scheme

Review proceeded agency by agency. Following an initial review of specific strategies employed in four behavior focused (NIAAA, NICHD, NIDA and NIMH) and four disease focused agencies (NCI, NIDDK, NHLBI and NIA), we developed a tentative list of categories that summarize the kinds of interventions that we noted in the abstract review process. When a subsequently reviewed grant had an approach that could not be easily accommodated within the existing list of categories, we added a new category. For each grant, the proposed categories were noted as present or not present. Single grants could include multiple categories. All grants included at least one category.

### Artificial Intelligence

In the context of smartphone apps, artificial intelligence implies the use of embedded computational algorithms that give the user guidance for making ideal or maximally satisfying decisions [43]. The user provides input either by defining situations and selecting options provided by the app or through data provided from wearable devices. The intent is to guide the user in taking next steps toward enhancing prevention or engaging in treatment [44]. Included in this category are apps that feature tailoring to meet an individual’s personalized needs.

### Bionic Adaptation

This category of apps included grants that intend to develop smartphone assisted or adaptive technologies and prosthetic devices to compensate for a variety of biological limitations or disease conditions. Conceivably, bionic adaptations can be used to compensate for challenges with vision [45,46], vestibular balance [47,48], speech and hearing [49,50], and the use of artificial limbs [51,52]. The goal is often to replace a missing component of normal human functioning or enhance a component that has been damaged due to aging, illness or injury. Included in this category are wearable and injected devices that communicate wirelessly with smartphones.

### Cognitive and Behavioral Therapies

This category includes a wide variety of interventions. Among these, cognitive behavior therapy (CBT) involves treatments that seek to change behavior by modifying how people process self-relevant information [53]. The underlying assumption is that people have dysfunctional thoughts and that these thoughts lead them to behave in maladaptive ways that can be corrected with supportive counseling. The CBT approach seeks to challenge a person’s assumptions and how they interpret events around them. The goal of CBT is to replace negative or self-defeating beliefs, thoughts, and feelings with positive and self-enhancing cognitions. Therapeutic treatments involving CBT typically train individuals to self-monitor and identify harmful thoughts as well as eliminate distorted cognitions (eg, unnecessary worry) that lead to dysfunctional behaviors. Core features include developing problem solving and coping strategies, anxiety reducing techniques, progressive relaxation, cognitive restructuring, activity scheduling, and other skills training approaches (eg, assertiveness) that reduce the influence of unhelpful thinking patterns. An added approach, behavioral activation (BA) [54] specifically addresses scheduling positive activities. We categorized grants as having cognitive and behavioral therapy components even if CBT or BA were not mentioned, but cognitive interventions falling under the CBT rubric were addressed.

### Contingency Management

This generally refers to interventions that provide overt consequences, typically in the form of rewards, when behavior standards are met [55,56]. Apps that address contingency management often provide some form of monetary reward. Using rewards is most common in cases where app users are required to forego behavior, such as in treating different forms of addiction [57], or users want to promote a behavior, such as physical exercise among sedentary individuals [58] or individuals recovering from surgery [59]. The provision of financial incentives (ie, the carrot and stick approach in behavioral economic theory), suggests that human beings are rational agents that will deliberate consciously when faced with a decision, and if offered a valued incentive, change their behavior [60-64].

### Education and Information

Apps in this category provide information and serve to educate patients about their disease, condition, or disorder. One of the primary aspects that characterize apps in this category is providing access to research on the disorder in a manner that is acceptable and understandable to a lay audience [65]. There may be a variety of reasons for providing this information, including increasing understanding of and countering misperceptions about the course of a disease [66-68], increasing beliefs about negative consequences of inaction or dysfunctional action [69,70] and increasing beliefs that taking proper action will be beneficial [70,71].

### Enhanced Motivation

This merits an umbrella classification because it involves interventions that attempt to increase a user’s inherent desire to behave in a manner that improves health behaviors. Included in this rubric is motivational interviewing (MI) [72-74] as well as a variety of other strategies. The focus of motivational enhancement is to increase a person’s desire to align their behaviors with their primary values and reduce their ambivalence through a focus on the pursuit of goal directed activities. This approach assumes that people can articulate their thinking about goals and aspirations. Aligning their behavior with goals, priorities and values helps individuals reduce cognitive dissonance [75]. Thus, instead of focusing on changing or developing new cognitions, motivational approaches capitalize on and strengthen existing cognitions. Apps that focus on motivation often focus on helping users remember their overarching life goals and engage in goal setting activities.

### Facilitating, Reminding, and Referring

These smartphone apps provide a means for users to connect with resource providers, primarily clinicians and testing facilities, and they address improving access to healthcare [76,77]. This can involve finding resources as well as providing reminders and scheduling alerts to assist users in remembering appointments, when to take medication [78] and finding relevant resources they need [79,80].

### Gaming and Gamification

This category of apps involves users in some form of imaginative (ie, virtual simulation) and competitive play [81-83]. The goal is to provide a basis for active learning and foster skill development. This can include stealth learning, where the user is not entirely aware of the game logic but acquires new cognitive skills while playing the game [84,85]. This is typically done irrespective of directly providing instruction. Rather, users are expected to learn through the experiences provided by the game’s design. Games include a variety of formats including timed challenges, simulations, and exercises in which users earn points (ie, rewards) through correct responses.

### Mindfulness Training

An alternative cognitive approach that encourages individuals to be introspective and pay attention to moment by moment experiences and heed one’s inner voice [86-89]. The goal of mindfulness training in smartphone apps is to heighten users’ awareness, encourage them to live in the moment and be aware of their surroundings in a more relaxed and contemplative state of mind [90]. Meditation is a core feature of mindfulness training, using deep relaxation and breathing exercises to accompany skills and strategies that target defeating negative thoughts and reducing temptations to overreact to nonessential stimuli.

### Monitoring and Feedback

This a broad catchall for apps that collect biological data (from sources such as saliva, blood or urine), activity data (from telemetry or accelerometer devices that detect movement or location) or detect vital signs (involving sensors), as well as self-report data (from ecological momentary assessments). Apps that are linked with wireless wearable devices or subcutaneous embedded sensors [91-93] are also included. In part, the value of monitoring is that, without adequate information, errors in diagnosis and treatment may occur [94]. Thus, there is a clear benefit to the clinician who intends to understand how the patient responds or acts when not present in the clinic.

A second value of monitoring is that it creates opportunities for clinic staff to keep patients and other non-clinical parties informed and provide them feedback. Many apps address monitoring as well as provide the app user feedback about their performance, such as adherence to medication and prescribed health promotion routines based on the monitoring just received. Indeed, without monitoring, feedback would not be possible, so it became a corollary activity of these apps. Research has demonstrated the potential that positive feedback can have on health outcomes [95,96].

### Norm Setting

These apps draw from the literature in which these norm setting approaches have been tried with adolescents [97] and college-age populations [98,99]. The hypothesis underlying these approaches is that many people overestimate the prevalence and acceptability of negative behaviors, such as alcohol and drug use. Interventions reduce risk by providing accurate information about the prevalence and unacceptability of behaviors among referent peer groups. Correcting misperceptions regarding social acceptability and prevalence also serves to modify expectancies about perceived benefits that underlie engaging in risky behavior.

### Skill Training

Apps in this category tend to focus on stress and emotional self-regulation. These apps provide cues on how to respond to challenging situations. Apps often focus on pain, anxiety, stress and emotion management [100,101]. Skills taught may also include how to be assertive and refuse unwanted offers to participate in risky behaviors [102]. These apps walk users through different strategies they can apply when handling challenging situations. Examples of skills frequently taught include general life skills such as decision making, goal setting and communication skills.

### Social Support and Social Networking

This category of apps provides ways to link a patient or app user to family and friends [103]. These apps may provide access to a network of individuals similar to the user who share similar health conditions and who can provide support and information. The implied effect by increasing support and including a user’s social network is that there will be both an empathetic response and the potential for those in the network to provide some type of instrumental support or assistance. Apps that fall under this broad rubric are in part driven by the literature linking social support with better health outcomes [104,105].

## Results

Based on the search criteria, a total of 1524 grants met the activity code requirements. After reading the abstract for each grant to verify that a smartphone intervention app was indeed proposed, and that enough detail was provided to categorize the intervention, the resulting qualifying pool included 397 grants in the final sample.

The most common activity codes represented were Research Project Grants (R01) (103 grants; 25.9%) and R43 Phase I Small Business Innovation Research (SBIR) grants (also 104 grants; 26.2%). Together SBIR (R43 and R44) and Small Business Technology Transfer (STTR) (R41 and R42) grants accounted for 162 (40.8%) of the grants. Two categories of exploratory grants, R21s (70 grants; 17.6%) and R34s (37 grants; 9.3%), were also prominent. R03 (10 grants), R15 (5 grants), R56 (5 grants), R61 (3 grants), R35 (1 grant) and R37 (1 grant) together accounted for 6.2% of the grants.

Table 1 summarizes the number of grants that met the selection criteria and shows the total first grant year dollars awarded for these grants for the years 2014 through 2018 (dollars are not adjusted for inflation.) Total funding for the first year of projects increased steadily and the percentage these grants represent doubled throughout the five-year period. While this represents a sizable investment in absolute dollars, funding for smartphone intervention apps was a miniscule part of overall NIH funding for the agencies that sponsored this research.

**Table 1:** Year of funding and one-year grant awards.

Year	Number of Grants	Total Dollars ($)	Average Grant ($)	Agency Funding, %
2014	60	16,783,541	279,726	0.06
2015	75	25,871,328	344,951	0.09
2016	69	26,493,472	383,963	0.09
2017	81	29,533,319	364,608	0.10
2018	112	39,428,610	352,041	0.12
Total	397	138,110,270	345,058	0.09

All NIH agencies reviewed, except for NHGRI, contributed projects to our sample. Table 2 presents the number of smartphone intervention app grants awarded by each NIH agency. NIA awarded a large number of grants (76). Several other agencies were also prolific in awarding grants for developing and testing smartphone intervention apps: NCI (47), NIMH (41), NIDDK (37), NIDA (31) and NHLBI (31). During any given year, the size of an agency’s budget was not significantly associated with how many smartphone intervention app grants were funded (*r*=.018).

The next step involved coding each grant’s abstract based on the 13 intervention categories. All grants could be coded based on single or multiple categories, should such evidence exist. In the 397 grant applications, the number of categories of intervention ranged from 1 to 5 with a mean of 1.99. Most grants proposed either one (139 grants, 35.0%) or two (155 grants, 39.0%) intervention approaches.

**Table 2:** Smartphone 1-year grant awards by NIH agency.

Agency	Grants	Grants Awarded, %	Total Funding ($)	Average Grant ($)
NIAAA	22	5.8	8,067,120	366,687
NIA	76	18.5	25,487,479	335,362
NIAID	6	1.8	2,509,169	418,195
NIAMS	4	0.6	862,077	215,519
NCI	46	11.8	16,363,863	355,736
NIDA	31	7.1	9,842,481	317,499
NIDCD	18	4.3	5,910,960	328,387
NIDCR	1	0.2	223,252	223,252
NIDDK	37	11.0	15,219,500	411,338
NIBIB	4	0.8	1,158,194	289,549
NIEHS	9	1.0	1,396,922	155,214
NEI	3	0.5	664,506	221,502
NIGMS	9	1.8	2,548,240	283,138
NICHD	15	3.4	4,657,284	310,486
NHLBI	31	11.1	15,283,691	493,022
NLM	3	0.6	777,482	259,161
NIMHD	21	3.7	5,144,671	244,984
NIMH	41	11.2	15,487,235	377,737
NINR	18	4.4	6,106,917	339,273
NINDS	2	0.3	399,227	199,614
Total	397	100.0	138,110,270	307,283

**Table 3:** Relative emphasis of intervention strategies in NIH-funded smartphone apps (N=397).

Content Category	Number of Apps	Total Apps, %	Rank
Artificial Intelligence	57	14.4	7
Bionic Adaptation	33	8.3	10
Cognitive and Behavioral Therapies	68	17.1	4
Contingency Management	24	6.0	11
Education and Information	85	21.4	3
Enhanced Motivation	50	12.6	9
Facilitating, Reminding and Referring	60	15.1	5
Gaming and Gamification	52	13.1	8
Mindfulness Training	18	4.5	12
Monitoring and Feedback	192	48.4	1
Norm Setting	7	1.8	13
Skills Training	85	21.4	2
Social Support and Social Networking	59	14.9	6

Table 3 summarizes the relative emphasis placed on the types of intervention approaches that were coded. By far, the most commonly included intervention approach provided monitoring and feedback to targeted individuals (48.4%). In 15.7% of cases, monitoring and feedback apps included some form of a wearable device that provided data that was transmitted through the smartphone. Also included were apps that used geo-sensors that could relay information about a user’s location as well as self-report functions. Contingency management that would involve some form of monetary reward or incentive was much less frequently observed but was always tied to monitoring. Providing education and information (21.4%) and separately providing skills training (21.4%) were each represented in about one in five proposed apps. Apps that intended to provide cognitive and behavior therapies (17.1%), facilitating, reminding and referring (15.1%), social support and opportunities for joining social networks (14.9%), enhancing a user’s motivation to improve their behaviors or comply with prescribed regimens (12.6%) and apps that proposed to use some form of game (13.1%) were also observed with some frequency. On the rare side were apps that focused on norm setting (1.8%) and mindfulness training (4.5%). Bionic adaptation apps that proposed linking smartphones to prosthetic devices were also relatively rare (8.3%).

## Discussion

### Principal Results

It is worth emphasizing that all abstracts represented proposed projects that had undergone rigorous peer review and been funded. NIH funds anywhere between 10% and 20% of submitted applications on an annual basis. Successfully competing for NIH funding therefore provides some assurance that the abstracts reviewed represent the state of the science.

Smartphone intervention apps are increasing in popularity among researchers being funded by NIH. The increase in numbers of grants funded between 2014 and 2018 attests to the belief in the potential for smartphone technology to be useful in health promotion and disease prevention. It is conceivable that this increase is due primarily to the increased technology capability that smartphones offer, their broad reach, ease of use by potential users, flexibility, and increasing ease of app programming.

The current review found that each NIH agency’s research agenda was matched with possible smartphone intervention apps that addressed their programmatic goals. The behaviorally focused agencies (eg, NIAAA, NICHD, NIDA and NIMH) funded grants that emphasized psychosocial interventions. However, disease focused agencies also included funding for smartphone intervention app projects that addressed behavioral concerns, notably focusing on monitoring and feedback. The diversity of app categories and intervention strategies was interesting given that we identified 13 discrete intervention categories in comparison to the four categories suggested by Abraham and Michie [106]. Their categories included: (1) adherence and remote monitoring; (2) remote dissemination of information; (3) data collection and disease outbreak surveillance; and (4) diagnostic treatment and support. Their classification scheme was specific to physical activity and dietary interventions and not extended to a broad variety of smartphone apps.

There are yet many approaches to intervention that are currently less robustly represented in the current taxonomy. For example, smartphones have significant potential to deliver interventions that can augment participant engagement through gamification. In the grants reviewed, gamification (13.1%) was most often included with grants that also addressed skills training (34.6%), monitoring and feedback (26.9%), and education and information (25.0%). Less frequently it was coupled with cognitive and behavioral therapies (17.3%), contingency management (11.5%), social support and social networking (11.5%), artificial intelligence (7.7%), bionic adaptation (5.8%), enhanced motivation (7.7%), mindfulness training (1.9%) and facilitating, reminding and referring (1.9%). It was never coupled with norm setting. This coupling of strategies may indicate the desire to utilize fun gaming logic with other active program ingredients. Conceivably, strategies could be more frequently coupled to more robustly utilize the gamification potential of smartphones.

Grant review has been noted as fostering conservatism in its outcomes [107], with relative ease in providing fundable scores to traditional topics and difficulty in assigning equally meritorious scores to novel approaches. When examined from this point of view, norm setting and mindfulness training were the least utilized approaches for interventions. Perhaps these may represent newer approaches for which researchers have not yet successfully competed for grant funding.

The creation of a categorization scheme is intended to provide a simplified structure by which topics covered can be grouped for analysis. NIH funded grants are truly multifarious, covering a wide array of diseases and conditions that are addressed through an equally diverse set of interventions. The categorization scheme presented reflects both our understanding of the underlying theoretical and practical assumptions investigators used when crafting their grant applications. The diversity of wording used in abstracts, even within a topic area, presents a challenge to anyone who would attempt to simplify an entire field. We note especially that there were many cases where abstracts were somewhat vague about the approach being proposed. Nonetheless, the resulting classification system provides an initial way in which intervention types may be characterized and meets the needs of our analysis.

### Limitations

NIH grant summaries and abstracts presented in NIH Reporter are the only freely available descriptions of grant applications that can be accessed without making a freedom of information request. They are abbreviated descriptions that, under current guidelines, are limited to 30 lines of text. As a result, they often lack sufficient detail that would otherwise be found in a grant’s research plan section. Because of this, it is likely that details about interventions in an abstract may be missing or underspecified. Nonetheless, abstracts provided enough information for the purpose of the current paper to construct a general picture of the state of the science in funded grants.

Our review was limited to examining funded grants and not the outcomes of these grants. Future research will need to examine the strategies proposed to determine if researchers’ planned interventions were successful at achieving intended results.

### Access

Our classification results are publicly available for download [108].

### Conclusion

Our review of NIH-funded abstracts over a five-year timeframe suggests that there is growing interest in using smartphone apps as either standalone or auxiliary components of health promotion and disease, as well as injury prevention and treatment. While smartphone intervention apps being developed with NIH grants are no longer novel, there is still great opportunity for innovation and rigorous science to provide a body of evidence-based strategies.

## Abbreviations

BA: behavioral activation
CBT: cognitive behavior therapy
eHealth: electronic health
GPS: global positioning system
mHealth: mobile health
MI: motivational interviewing
NEI: National Eye Institute
NHGRI: National Human Genome Research Institute
NHLBI: National Heart, Lung, and Blood Institute
NIA: National Institute on Aging
NIAAA: National Institute on Alcohol Abuse & Alcoholism
NIAID: National Institute of Allergy and Infectious Diseases
NIAMS: National Institute of Arthritis and Musculoskeletal and Skin Diseases
NIBIB: National Institute of Biomedical Imaging and Bioengineering
NICHD: National Institute of Child Health and Human Development
NIDA: National Institute on Drug Abuse
NIDCD: National Institute on Deafness and other Communication Disorders
NIDCR: National Institute of Dental and Craniofacial Research
NIDDK: National Institute of Diabetes and Digestive and Kidney Diseases
NIEHS: National Institute of Environmental and Health Sciences
NIGMS: National Institute of General Medicine Sciences
NIH: National Institutes of Health
NIMH: National Institute of Mental Health
NIMHD: National Institute on Minority Health and Health Disparities
NINDS: National Institute of Neurological Disorders and Stroke
NINR: National Institute of Nursing Research
NLM: National Library of Medicine
VoIP: Voice over Internet Protocol
